# Screening for tuberculosis among high-risk groups attending London emergency departments: a prospective observational study

**DOI:** 10.1183/13993003.03831-2020

**Published:** 2021-06-24

**Authors:** Rishi K. Gupta, Swaib A. Lule, Maria Krutikov, Lara Gosce, Nathan Green, Jo Southern, Ambreen Imran, Robert W. Aldridge, Heinke Kunst, Marc Lipman, William Lynn, Helen Burgess, Asif Rahman, Dee Menezes, Ananna Rahman, Simon Tiberi, Peter J. White, Ibrahim Abubakar

**Affiliations:** 1 Institute for Global Health, University College London, London, UK; 2 MRC Centre for Global Infectious Disease Analysis and NIHR Health Protection Research Unit in Modelling and Health Economics, Imperial College London, London, UK; 3 TB Unit, Public Health England, Colindale, London, UK; 4 Royal Free London NHS Foundation Trust, London, UK; 5 Centre for Public Health Data Science, Institute of Health Informatics, University College London, London, UK; 6 Blizard Institute, Queen Mary University of London, London, UK; 7 UCL-TB and UCL Respiratory, University College London, London, UK; 8 London North West University NHS Trust, London, UK; 9 West Middlesex University Hospital, Chelsea and Westminster NHS Foundation Trust, London, UK; 10 Imperial College London NHS Trust, London, UK; 11 Division of Infection, Barts Health NHS Trust, London, UK; 12 Modelling and Economics Unit, National Infection Service, Public Health England, London, UK

## Abstract

Most tuberculosis (TB) cases in low-incidence settings are thought to be due to reactivation of latent TB infection (LTBI) in high-risk populations [1–3]. Assessment of patients at emergency departments (EDs) is a potential opportunity to achieve early TB diagnosis, and interrupt transmission. An earlier study in London found that 39% of patients diagnosed with TB had attended an ED in the preceding 6 months [4]. Of these, 76% had a chest radiograph performed, of which 86% and 40% were abnormal in cases of pulmonary and extrapulmonary TB, respectively. Attendance at EDs provides an opportunity to identify individuals with LTBI, who may be at risk for progression to active disease and unlikely to engage with healthcare services *via* other routes.

## Abstract 2

**LTBI screening among high-risk groups at EDs could be implemented to identify those at risk of progression to TB disease. Large-scale studies are required to investigate effective TB disease screening strategies in EDs.** https://bit.ly/3bTkoOn

*To the Editor:*

Most tuberculosis (TB) cases in low-incidence settings are thought to be due to reactivation of latent TB infection (LTBI) in high-risk populations [1–3]. Assessment of patients at emergency departments (EDs) is a potential opportunity to achieve early TB diagnosis, and interrupt transmission. An earlier study in London found that 39% of patients diagnosed with TB had attended an ED in the preceding 6 months [4]. Of these, 76% had a chest radiograph performed, of which 86% and 40% were abnormal in cases of pulmonary and extrapulmonary TB, respectively. Attendance at EDs provides an opportunity to identify individuals with LTBI, who may be at risk for progression to active disease and unlikely to engage with healthcare services *via* other routes.

Between July 2013 and May 2017, we recruited individuals over the age of 16 years, who were recent entrants from, or prolonged travellers to, high TB incidence countries, or people with a history of homelessness, imprisonment or problem drug use attending EDs at seven London hospitals. We investigated the yield of interferon-γ release assay (IGRA) and TB disease screening among eligible ED attendees, regardless of their reason for ED attendance. Participants were tested with either the QuantiFERON Gold-in-Tube (Qiagen, Hilden, Germany), or QuantiFERON-TB Plus. A subset of participants was screened for TB disease; symptomatic individuals (≥2 week history of cough or fever, accompanied by haemoptysis, drenching night sweats, or unexplained weight loss) underwent chest radiograph, sputum Xpert MTB/RIF testing and subsequent referral for further evaluation and management [5]. Those screened with IGRA were followed-up through data linkage to national TB surveillance notifications to identify subsequent active TB notification [6]. This study was approved by the Stanmore National Health Service Research Ethics Committee (14/LO/2160) and registered on ClinicalTrials.gov (NCT02512484; full study protocol available at the institutional website [7]).

Descriptive analyses were performed to assess the yield of screening and incidence rates of TB disease among those tested by IGRA. Logistic regression models were used to examine factors associated with IGRA positivity. The final multivariable model included *a priori* variables (age, sex, presence of social risk factors, history of TB contact, ethnicity and country of birth), and variables found to be significant in univariable analyses (p<0.2).

A total of 1407 participants were recruited to the IGRA screening study, of whom 241 (17.1%) had a history of substance use disorders, homelessness or imprisonment, while the remainder were migrants from high TB burden countries. Of the 1407 participants, 642 (45.6%) were female, and the majority (1010; 70.8%) were over 35 years of age (median 45 years). Among those recruited due to recent migration from, or travel to, a high TB incidence country, most participants (736/1166; 63.1%) were South Asian, while the largest ethnic group among those with social risk factors was white (100/241; 41.5%). Almost one fifth (258/1407; 18.2%) of participants reported previous contact with a TB case. Diabetes was common, affecting 239/1407 (17.0%). Of the 1407 participants, 109 (7.7%) were not registered with a general practitioner; this was more common among those with social risk factors (35/241; 14.5%) when compared with the migrant group (74/1166; 6.3%).

IGRA results were available for 1232 participants, of which 34 (2.4%) were indeterminate. A total of 256/1198 (21.4%) participants with valid available results were IGRA positive. The prevalence of IGRA positivity was 24% among migrants and 19% among those with social risk factors (table 1). In a multivariable logistic regression model, only male sex (OR 1.38, 95% CI 1.01–1.87; p=0.041), age >35 years (OR 1.67, 95% CI 1.12–2.55; p=0.012) and non-UK country of birth (OR 6.32, 95% CI 2.99–15.6; p<0.001) were independently associated with IGRA positivity.

**TABLE 1:** Factors associated with interferon-γ release assay (IGRA) positivity in univariable and multivariable logistic regression models

**Characteristic**	**IGRA result**		**Univariable**				**Multivariable**		
	**Negative (n=942)**	**Positive (n=256)**	**Subjects n**	**OR**	**95% CI**	**p-value**	**OR**	**95% CI**	**p-value**
**Age years**			1193			<0.001			0.012
≤35	286 (87%)	41 (13%)							
>35	652 (75%)	214 (25%)		2.29	1.61–3.33		1.67	1.12–2.55	
Missing	4	1							
**Sex**			1193			0.062			0.041
Female	446 (81%)	104 (19%)							
Male	493 (77%)	150 (23%)		1.30	0.99–1.73		1.38	1.01–1.87	
Missing	3	2							
**Ethnicity**			1188			0.007			0.2
White	165 (87%)	24 (13%)							
South Asian	534 (77%)	162 (23%)		2.09	1.34–3.38		1.56	0.89–2.89	
Black African or Caribbean	52 (73%)	19 (27%)		2.51	1.27–4.95		2.13	0.96–4.75	
Other	184 (79%)	48 (21%)		1.79	1.06–3.10		1.29	0.68–2.51	
Missing	7	3							
**Country of birth**			1190			<0.001			<0.001
UK	180 (93%)	13 (6.7%)							
Non-UK	755 (76%)	242 (24%)		4.44	2.58–8.33		6.32	2.99–15.6	
Missing	7	1							
**TB contact**	161 (75%)	53 (25%)	1160	1.28	0.89–1.80	0.175	1.28	0.87–1.85	0.2
Missing	29	9							
**Diabetes**	147 (74%)	53 (26%)	1191	1.40	0.98–1.98	0.064	1.16	0.79–1.68	0.4
Missing	7	0							
**Any social risk factor^#^**	153 (81%)	37 (19%)	1198	0.87	0.58–1.27	0.483	1.20	0.70–2.01	0.5
**BMI kg·m^-2^**			1165			0.032			0.5
≤25	398 (82%)	90 (18%)							
>25	517 (76%)	160 (24%)		1.37	1.03–1.83		1.10	0.81–1.51	
Missing	27	6							
**Travel (past 3 years)^¶^**	809 (78%)	234 (22%)	1177	1.86	1.14–3.22	0.012	1.35	0.67–2.91	0.4
Missing	17	4							
**Registered with a GP**	871 (78%)	240 (22%)	1198	1.22	0.72–2.21	0.474			

Data shown for participants with valid IGRA results (n=1198). n=1027 in multivariable model. Variables were included in the model if considered of clinical importance *a priori*, or if significant in univariate analysis (p<0.2). Percentages reflect row percentages. TB: tuberculosis; BMI: body mass index; GP: general practitioner. ^#^: includes history of homelessness, imprisonment or harmful drug use; ^¶^: indicates any travel to a high TB incidence country in the past 3 years.

Participants screened by IGRA were followed up for a median of 381 days (interquartile range 303–605 days), *via* linkage to national TB surveillance records until 31 December 2017. Of the 256 with a positive IGRA, five were notified with TB disease during follow-up, giving a TB incidence rate among those with a positive IGRA of 1476.4/100 000 person-years (95% CI 529.4–3173.2). All five TB cases had extrapulmonary disease (lymph node (notified 5 days after recruitment), spinal (35 days), disseminated (52 days), genito-urinary (162 days) and intra-abdominal (412 days)), and none reported recent TB contact at study recruitment. Median quantitative interferon-γ responses to *Mycobacterium tuberculosis* antigens were 6.02 IU·mL^−1^ (range 0.62 to >10 IU·mL^−1^) among the five progressors to TB disease, compared to 2.57 IU·mL^−1^ (0.35 to >10 IU·mL^−1^) among non-progressors. No TB cases were notified among participants who had negative or indeterminate IGRAs.

Of the 513 participants screened in the active TB study, only 14 (2.7%) were symptomatic. Of these, 13 had a chest radiograph, with eight providing an adequate sputum sample for Xpert MTB/RIF testing. None of these patients were diagnosed with TB during the study.

Previous studies have retrospectively reviewed ED presentations among notified TB cases in low-incidence settings [4, 8–11], and examined the yield of TB screening in high-incidence countries [12, 13]. A study in the USA also investigated the yield of LTBI screening among risk groups attending EDs using the tuberculin skin test (TST) [14]. Notably, nearly half of the participants in the US study did not return to have the TST read. Our study has demonstrated the feasibility of prospective IGRA testing among high-risk groups attending EDs in London. Valid IGRA results were available for 85% of participants, which is comparable to other IGRA evaluations [15, 16]. Linkage to national TB surveillance records provided a previously validated mechanism to identify participants screened by IGRA who subsequently progressed to TB disease [6], with median follow-up longer than 1 year.

Major study limitations were that patients with positive IGRA results were not routinely linked to TB services for evaluation for TB disease and consideration of LTBI treatment, since LTBI screening among risk groups attending EDs was not part of national policy at the time. Thus, we are unable to estimate the proportions of IGRA-positive participants who would have started and completed preventative therapy if referred to TB services following detection. This remains a key knowledge gap, since effectiveness (and cost-effectiveness) of screening is dependent upon completion of LTBI treatment for these individuals, to reduce risk of TB disease. In addition, although the vast majority of participants in the study were registered with general practitioners, it is unclear how engaged they were with these services, limiting our ability to assess precisely how well ED screening could complement the primary care screening programme. Data were not available on individuals who attended EDs and were screened for eligibility but not recruited to the study.

In the TB disease screening study arm, we proactively screened for pulmonary TB disease using a symptom screen followed by chest radiograph and sputum testing. Consequently, pulmonary TB cases without typical symptoms and extrapulmonary cases may have been missed. Moreover, our sample size of 513 participants screened for TB symptoms may have been too small to detect active cases; we therefore could not evaluate risk factors for active TB or cost-effectiveness of TB disease screening.

In summary, our study suggests that ED IGRA screening among TB risk groups could be implemented to identify individuals at risk of TB who may be difficult to engage *via* other screening approaches. Such screening must be supported by a dedicated protocol that detects individuals at higher risk of TB for screening, and resources to facilitate onward referral of those with a positive IGRA to local TB services. In contrast, this study suggests that resource-intensive, symptom-based active TB screening in EDs is unlikely to be worthwhile as the yield is likely to be too low to justify the resource. However, other high-throughput screening models such as automated, routine chest radiograph review in EDs may be evaluated in future research.

## Shareable PDF

10.1183/13993003.03831-2020.Shareable1 This one-page PDF can be shared freely online.
Shareable PDF ERJ-03831-2020.Shareable
