# Supplementary material for: Screening for tuberculosis among high-risk groups attending London emergency departments: a prospective observational study
Source: Eur Respir J. 2021 Jun 24;57(6):2003831. doi: 10.1183/13993003.03831-2020 (PMC8223173; doi:10.1183/13993003.03831-2020)
Supplement: Supplementary file 1 [file ERJ-03831-2020.Shareable.pdf]

AGORA
RESEARCH LETTER

SHAREABLE PDF

Screening for tuberculosis among high-risk groups attending London emergency departments: a prospective observational study

Rishi K. Gupta [1], Swaib A. Lule[1], Maria Krutikov [1], Lara Gosce [1], Nathan Green[2], Jo Southern[3], Ambreen Imran [4], Robert W. Aldridge [5], Heinke Kunst[6], Marc Lipman [4,7], William Lynn[8], Helen Burgess[9], Asif Rahman[10], Dee Menezes[5], Ananna Rahman[6], Simon Tiberi[6,11], Peter J. White[2,12] and Ibrahim Abubakar [1]

Affiliations: [1]Institute for Global Health, University College London, London, UK. [2]MRC Centre for Global Infectious Disease Analysis and NIHR Health Protection Research Unit in Modelling and Health Economics, Imperial College London, London, UK. [3]TB Unit, Public Health England, Colindale, London, UK. [4]Royal Free London NHS Foundation Trust, London, UK. [5]Centre for Public Health Data Science, Institute of Health Informatics, University College London, London, UK. [6]Blizard Institute, Queen Mary University of London, London, UK. [7]UCL-TB and UCL Respiratory, University College London, London, UK. [8]London North West University NHS Trust, London, UK. [9]West Middlesex University Hospital, Chelsea and Westminster NHS Foundation Trust, London, UK. [10]Imperial College London NHS Trust, London, UK. [11]Division of Infection, Barts Health NHS Trust, London, UK. [12]Modelling and Economics Unit, National Infection Service, Public Health England, London, UK.

Correspondence: Ibrahim Abubakar, Institute for Global Health, University College London, 30 Guilford Street, London, UK. E-mail: i.abubakar@ucl.ac.uk

This study is registered at ClinicalTrials.gov (NCT02512484). The authors agree to share the data on reasonable request.

@ERSpublications
LTBI screening among high-risk groups at EDs could be implemented to identify those at risk of progression to TB disease. Large-scale studies are required to investigate effective TB disease screening strategies in EDs. https://bit.ly/3bTkoOn

Cite this article as: Gupta RK, Lule SA, Krutikov M, *et al*. Screening for tuberculosis among high-risk groups attending London emergency departments: a prospective observational study. *Eur Respir J* 2021; 57: 2003831 [https://doi.org/10.1183/13993003.03831-2020].

This single-page version can be shared freely online.

To the Editor:

Most tuberculosis (TB) cases in low-incidence settings are thought to be due to reactivation of latent TB infection (LTBI) in high-risk populations [1–3]. Assessment of patients at emergency departments (EDs) is a potential opportunity to achieve early TB diagnosis, and interrupt transmission. An earlier study in London found that 39% of patients diagnosed with TB had attended an ED in the preceding 6 months [4]. Of these, 76% had a chest radiograph performed, of which 86% and 40% were abnormal in cases of pulmonary and extrapulmonary TB, respectively. Attendance at EDs provides an opportunity to identify individuals with LTBI, who may be at risk for progression to active disease and unlikely to engage with healthcare services *via* other routes.

Copyright ©The authors 2021.. This version is distributed under the terms of the Creative Commons Attribution Licence 4.0.
